# Cardiovascular comorbidities and cancer-directed therapies in Hispanic breast cancer patients: a two-center analysis from the Southwestern U.S

**DOI:** 10.3389/fonc.2025.1637171

**Published:** 2025-09-17

**Authors:** Tomas Escobar Gil, Emily Sherry, Oscar Felipe Borja Montes, Alexandra Claire Millhuff, Valeria Guadalupe Hanson, Victoria Ayodele, Aymen Baig, Jacklyn Marie Nemunaitis, Marcela Mazo Canola

**Affiliations:** ^1^ Department of Internal Medicine, University of New Mexico Health Sciences Center, Albuquerque, NM, United States; ^2^ Department of Internal Medicine, University of Texas Health Science Center San Antonio, San Antonio, TX, United States; ^3^ School of Medicine, University of Texas Health Science Center San Antonio, San Antonio, TX, United States; ^4^ Department of Medical Oncology, University of New Mexico Comprehensive Cancer Center, Albuquerque, NM, United States; ^5^ Department of Medical Oncology, University of Texas (UT) Health San Antonio Mays Cancer Center, San Antonio, TX, United States

**Keywords:** breast neoplasms, Hispanic, cardiotoxicity, hypertension, disparities

## Abstract

**Background:**

Cardiovascular disease (CVD) is a leading cause of mortality among breast cancer survivors, particularly affecting Hispanic women due to a high burden of comorbidities and treatment-related toxicities. However, real-world cardiovascular risk and treatment patterns in this population remain under-characterized.

**Methods:**

We conducted a retrospective review of 394 Hispanic patients with stage I–III breast cancer treated with curative intent between 2022 and 2023 at two institutions in the Southwestern U.S. Data included demographics, cancer therapy, cardiovascular comorbidities, and medication use.

**Results:**

The cohort had a mean age of 59.9 years and a mean BMI of 30.1 kg/m². Cardiovascular comorbidities were present in 57.5% of patients, which appears numerically higher than rates reported in prior breast cancer cohorts (~40%). Hypertension (45.7%) and diabetes (24.3%) also appeared numerically more common than prior national estimates. Among hypertensive patients, 73.9% were receiving antihypertensives, which is numerically lower than previously published rates (~77%). In contrast, 94.2% of patients with hyperlipidemia were on statins, numerically higher than prior reports (~70%). All patients with CVD were receiving aspirin. Chemotherapy was administered to 66% of the cohort, a numerically higher rate than prior Hispanic breast cancer studies (~48%). Anthracycline use (19.2%) aligned with national de-escalation trends.

**Conclusion:**

Hispanic patients with breast cancer in the Southwestern U.S. face a high burden of cardiovascular disease and numerically lower rates of antihypertensive use. These findings support the need for regionally tailored, integrated cardio-oncology approaches.

## Introduction

In 2024, approximately 43,700 women in the U.S. are expected to die from breast cancer (1). This disease remains a leading cause of cancer-related mortality, especially among Hispanic women, who face unique risks, including higher rates of cardiotoxicity from treatments such as anthracyclines and trastuzumab (2). Data show that nearly 40% of breast cancer patients are diagnosed with cardiovascular comorbidities, such as hypertension and heart failure, which can be exacerbated by treatment-related toxicities (1, 3). Specifically, cardiovascular disease is now a leading cause of death among women diagnosed with breast cancer, with studies indicating that approximately 30% of breast cancer survivors die from cardiovascular-related causes (4). In a cohort study, 12.8% of women undergoing breast cancer treatment were reported to have a history of cardiovascular disease at diagnosis (5). Health disparities contribute to these outcomes, with Hispanic women facing barriers to care and a slower decline in cancer-related fatalities compared to non-Hispanic White women (6, 7). Our study investigates these factors specifically within the Southwestern U.S., where the burden of breast cancer is significant, particularly among Hispanics in this region (8).

## Methods

We conducted a retrospective cohort study of 394 self-identified Hispanic patients with stage I–III breast cancer who received treatment with curative intent at two academic cancer centers in the Southwestern United States: The University of New Mexico Comprehensive Cancer Center (UNMCCC) and the UT Health San Antonio Mays Cancer Center (UTHSCSA). The study period spanned from January 1, 2022, to December 31, 2023.

Eligible patients were ≥18 years old, identified as Hispanic in the electronic health record, and had histologically confirmed stage I–III breast cancer. Patients with incomplete medical records or those treated with palliative intent were excluded. The final analytic cohort included 195 patients from UNMCCC and 199 from UTHSCSA.

Data were abstracted from institutional electronic medical records—Cerner PowerChart (UNMCCC) and Epic (UTHSCSA)—by trained clinical researchers using a standardized REDCap database. Collected variables included demographics (age, sex, BMI, race), tumor characteristics (stage, receptor subtype), treatment modalities (chemotherapy, radiation, immunotherapy, HER2-targeted therapy), and comorbid cardiovascular conditions.

Cardiovascular comorbidities were defined based on either a documented diagnosis in the medical record or active use of related medications during cancer treatment. These included hypertension, hyperlipidemia, diabetes, myocardial infarction, heart failure, arrhythmias, stroke, cardiomyopathy, and vascular disease. Medication use data included antihypertensives, statins, and aspirin. Chemotherapy administration was defined as receipt of at least one cycle during the study period.

The primary outcomes were prevalence of cardiovascular comorbidities and use of cardioprotective medications. Secondary outcomes included institution-level variations in treatment patterns, including chemotherapy regimen, BMI distribution, and cardiovascular medication use.

Data were abstracted using REDCap electronic data capture tools hosted at the University of New Mexico and the University of Texas Health San Antonio (RRID: SCR_003445). Statistical analyses were conducted using IBM SPSS Statistics (version 28; RRID: SCR_002865). No core facilities, cell lines, antibodies, organisms, or external databases requiring RRIDs were used in this study. Descriptive statistics were used to summarize patient characteristics and treatment variables. Categorical variables were reported as counts and percentages; continuous variables were summarized using means and standard deviations.

This study was designed as a descriptive analysis to characterize cardiovascular comorbidities and treatment patterns among Hispanic breast patients with breast cancer. No hypothesis testing, comparative analyses, or multivariable modeling were performed, as the primary objective was to provide descriptive, real-world data rather than infer causality.

## Results

The study included 394 self-identified Hispanic patients diagnosed with breast cancer. Of these, 195 patients were treated at the University of New Mexico Comprehensive Cancer Center (UNMCCC) and 199 at the UT Health San Antonio Mays Cancer Center (UTHSCSA) (**Table 1**). Men represented 1% of the cohort, with only four male patients at UNMCCC. The average age was 59.94 years (SD=12.11), and the overall mean body mass index (BMI) was 30.12 kg/m² (SD=6.58). Patients at UNMCCC had a mean BMI of 29.25 kg/m² (SD=6.62), while those at UTHSCSA had a mean BMI of 31.00 kg/m² (SD=6.45). Based on BMI classification, 38.9% of patients were normal weight (BMI < 25), 40.9% were overweight (BMI 25–29.9), and 20.3% were classified as obese (BMI ≥ 30). The above reflects a right-skewed distribution, with many patients clustered just below the obesity threshold.

**Table 1:** Demographic and treatment characteristics by institution.

Characteristic	UNMCCC	UTHSCSA
Patients (Total)	195	199
Mean Age (years)	59.94 (SD=12.11)	59.94 (SD=12.11)
Mean BMI (kg/m²)	29.25 (6.62)	31.00 (6.45)
HR+ Breast Cancer	161 (41.0%)	154 (39.1%)
Triple-Negative Breast Cancer	35 (8.9%)	23 (5.8%)
HER2+ Breast Cancer	42 (10.7%)	34 (8.6%)
Anthracycline Use	37 (9.3%)	39 (9.9%)
HER2-Directed Therapy	32 (8.1%)	28 (7.1%)
Immunotherapy	31 (7.8%)	6 (1.5%)
No Chemotherapy	106 (26.8%)	135 (34.2%)
Radiation Therapy	114 (58.5%)	104 (53.3%)

While all patients identified as Hispanic/Latino, 84.1% self-identified racially as Latino (White), with small numbers reporting other racial identities or declining to state. Most patients were diagnosed with hormone receptor-positive disease (80.1%), followed by triple-negative (14.7%) and HER2-positive (19.2%) subtypes. Chemotherapy was administered to 66% of the total cohort, with 19.2% receiving anthracyclines and 15.3% receiving HER2-targeted therapies. Immunotherapy was used in 9.4% of patients. A notable percentage of patients (34.2% at UTHSCSA and 26.8% at UNMCCC) did not receive chemotherapy. Radiation therapy was administered to 58.5% of patients across both institutions.

In terms of cardiovascular health (**Table 2**), 57.51% of patients had at least one cardiovascular comorbidity. Hypertension was the most common condition, affecting 44.1% at UNMCCC and 47.2% at UTHSCSA. Diabetes was reported in 24.3% of patients overall. Additional conditions included hyperlipidemia (30.7%), myocardial infarction (3.8%), heart failure (4.3%), arrhythmias (3.6%), stroke (4.3%), cardiomyopathy (2.0%), and vascular disease (5.1%).

**Table 2:** Cardiovascular comorbidities by institution.

Comorbidity	UNMCCC	UTHSCSA
Hypertension	86 (44.1%)	94 (47.2%)
Myocardial Infarction	9 (4.6%)	6 (3.0%)
Heart Failure	8 (4.1%)	9 (4.5%)
Diabetes	47 (24.1%)	49 (24.6%)
Arrhythmias	11 (5.6%)	3 (1.5%)
Stroke	3 (1.5%)	14 (7.0%)
Cardiomyopathy	4 (2.1%)	4 (2.0%)
Vascular Disease	13 (6.7%)	7 (3.5%)
Hyperlipidemia	58 (29.7%)	63 (31.7%)

Of the 180 patients with hypertension, 133 (73.9%) were on antihypertensive treatment (**Table 3**). Among the 121 patients with hyperlipidemia, 114 (94.2%) were on statin therapy (**Table 3**). All 53 patients with a documented history of myocardial infarction, stroke, or vascular disease were receiving aspirin. The use of cardioprotective medications (**Table 3**) varied between institutions, with numerically higher beta-blocker and mineralocorticoid use at UNMCCC, and higher GLP-1 and statin use at UTHSCSA.

**Table 3:** Cardioprotective medication use by institution.

Medication class	UNMCCC	UTHSCSA
Aspirin	18 (10.4%)	35 (10.4%)
ACE Inhibitors/ARBs	57 (22.6%)	76 (22.6%)
Beta-Blockers	50 (19.2%)	25 (7.5%)
Mineralocorticoids	1 (0.4%)	9 (2.7%)
Statins	40 (15.4%)	74 (22.0%)
SGLT2 Inhibitors	1 (0.4%)	5 (1.5%)
GLP-1 Agonists	8 (3.1%)	18 (5.3%)
No Cardio Meds	109 (42.1%)	70 (20.8%)

## Discussion

This two-center retrospective analysis highlights the substantial burden of cardiovascular comorbidities in Hispanic patients with breast cancer in the Southwestern United States. Our study extends this body of work by focusing exclusively on Hispanic patients in the Southwestern U.S., a region with documented disparities but limited published cardio-oncology data (9–13). Prior studies have reported cardiovascular comorbidity rates of approximately 40% in breast cancer populations (5); in this cohort, 57.5% of patients were affected, suggesting a numerically higher prevalence. Hypertension and diabetes were also numerically more common than in previously published Hispanic or general breast cancer cohorts (~43% and ~17%, respectively) (4). These findings underscore the importance of evaluating cardiovascular risk as part of comprehensive breast cancer care in this population.

A lower proportion (73.9%) of antihypertensive use among patients with hypertension was observed when compared to prior reports (3–5, 9). One example is the study from Jabbal et al. which sets the proportion to around 77% (4). In contrast, statin use was high (94.2%), exceeding prior general population estimates (~70%) (4). This may reflect institutional efforts to manage lipid profiles more aggressively or greater attention to secondary prevention strategies. The variability in cardioprotective medication use between institutions also suggests differences in local practice patterns, access to primary care, or patient adherence.

Chemotherapy was administered to 66% of the cohort, which is numerically higher than previous studies in Hispanic populations reporting treatment rates near 48% (2). This may reflect a younger average age, greater access to curative-intent therapy, or institutional efforts to ensure treatment adherence. Anthracycline use (19.2%) was consistent with national trends toward de-escalation due to known cardiotoxicity (3), particularly in patients with preexisting cardiovascular risk factors.

Institutional differences were observed in both cardiovascular risk profiles and treatment strategies. For example, UTHSCSA patients had a numerically higher mean BMI and lower chemotherapy rates compared to those at UNMCCC. These differences may reflect differences in patient mix or institutional practice, though causality cannot be inferred (6, 7).

The findings highlight the complex interplay between cancer care and cardiovascular health in Hispanic patients. Given the increasing awareness of cardiovascular disease as a leading cause of death in breast cancer survivors (1), and the unique barriers faced by Hispanic communities—including language, insurance access, and proximity to specialty care (6) —there is a clear need for more integrated cardio-oncology strategies. While this study is limited by its retrospective design and lack of long-term outcomes, it emphasizes the importance of identifying real-world Our study was intentionally designed as descriptive, without formal statistical comparisons between institutions or subgroups, to avoid overstating associations in the absence of confounder adjustment. Non-Hispanic patients were not included, limiting contextualization of findings. Additional limitations include potential misclassification of comorbidities from diagnosis codes and medication records, the retrospective design with missing data and unmeasured confounders, the short study period (2022–2023), which precludes assessment of long-term outcomes, and the absence of certain clinical variables such as cumulative anthracycline dose, endocrine therapy use, surgery rates, stage distribution, laterality, and smoking or alcohol history. Finally, results may not generalize beyond Hispanic populations in the Southwestern U.S. or outside academic cancer centers.

Future studies should evaluate longitudinal cardiovascular outcomes and test care models designed to improve coordination between oncology and cardiology services. Additionally, interventions to enhance medication adherence and access to preventive care may reduce long-term morbidity in this growing and underserved patient population.

## Conclusion

This research reveals the high rate of cardiovascular comorbidities in a predominantly middle-aged Hispanic female cohort with majority hormone receptor-positive breast cancer. The findings underscore the importance of understanding demographic and clinical characteristics to tailor interventions. Future studies should focus on expanding data collection and examining the relationships between treatment regimens and cardiovascular health outcomes to better care for Hispanic patients with breast cancer. Designing programs tailored for this specific population could be beneficial in achieving these goals.

## Data Availability

The raw data supporting the conclusions of this article will be made available by the authors, without undue reservation.

## References

[1] Siegel RL Giaquinto AN Jemal A. Cancer statistics, 2024. CA Cancer J Clin. (2024) 74:12–49. doi: 10.3322/caac.21820, PMID: 38230766

[2] Power EJ Chin ML Haq MM. Breast cancer incidence and risk reduction in the hispanic population. Cureus. (2018) e2235. doi: 10.7759/cureus.2235, PMID: 29713580 PMC5919763

[3] Papageorgiou C Andrikopoulou A Dimopoulos MA Zagouri F. Cardiovascular toxicity of breast cancer treatment: an update. In: Cancer Chemotherapy and Pharmacology, vol. 88. Springer Science and Business Media Deutschland GmbH, Heidelberg, Germany (2021). p. 15–24., PMID: 33864486 10.1007/s00280-021-04254-w

[4] Jabbal IS Dwivedi A Bilani N Dominguez B Botrus G Nahleh Z. Disparities in metabolic conditions and cancer characteristics among hispanic women with breast cancer: A multi-institutional study. Cancers (Basel). (2022) 14. doi: 10.3390/cancers14143411, PMID: 35884473 PMC9317401

[5] Hill DA Friend S Lomo L Wiggins C Barry M Prossnitz E. Breast cancer survival, survival disparities, and guideline-based treatment. Breast Cancer Res Treat. (2018) 170:405–14. doi: 10.1007/s10549-018-4761-7, PMID: 29569018 PMC6002943

[6] Yedjou CG Sims JN Miele L Noubissi F Lowe L Fonseca DD. Health and Racial Disparity in Breast Cancer. In: Advances in Experimental Medicine and Biology. Springer New York LLC, Heidelberg, Germany (2019). p. 31–49. 10.1007/978-3-030-20301-6_3 PMC6941147 31456178

[7] Lopez R Agullo P Lakshmanaswamy R. Links between obesity, diabetes and ethnic disparities in breast cancer among Hispanic populations. Obes Rev. (2013) 14:679–91. doi: 10.1111/obr.12030, PMID: 23611507

[8] Moore JX Royston KJ Langston ME Griffin R Hidalgo B Wang HE. Mapping hot spots of breast cancer mortality in the United States: place matters for Blacks and Hispanics. Cancer Causes Control. (2018) 29:737–50. doi: 10.1007/s10552-018-1051-y, PMID: 29922896 PMC6301114

[9] Hu Q Chang CP Rowe K Snyder J Deshmukh V Newman M. Disparities in cardiovascular disease risk among hispanic breast cancer survivors in a population-based cohort. JNCI Cancer Spectr. (2021) 5. doi: 10.1093/jncics/pkab016, PMID: 33889806 PMC8052955

[10] Maras AF Penedo FJ Ramirez AG Worch SM Ortiz MS Yanez B. Cardiometabolic comorbidities in Hispanic/Latino cancer survivors: prevalence and impact on health-related quality of life and supportive care needs. Supportive Care Cancer. (2023) 31:711. doi: 10.1007/s00520-023-08181-9, PMID: 37982906 PMC11302055

[11] Zhu C Shi T Jiang C Liu B Baldassarre LA Zarich S. Racial and ethnic disparities in all-cause and cardiovascular mortality among cancer patients in the U.S. JACC CardioOncol. (2023) 5:55–66. doi: 10.1016/j.jaccao.2022.10.013, PMID: 36875907 PMC9982284

[12] Vo JB Ramin C Lawrence WR Barac A Ho KL Rhee J. Racial and ethnic disparities in treatment-related heart disease mortality among US breast cancer survivors. JNCI Cancer Spectr. (2023) 7. doi: 10.1093/jncics/pkad024, PMID: 36943362 PMC10130190

[13] Sharma A Fierro ME Governor S Kothare A Pak S Liu K. Incidence and risk factors of trastuzumab-induced cardiac dysfunction in a predominantly Hispanic South Texas population: a descriptive study. Cardio-Oncology. (2025) 11. doi: 10.1186/s40959-025-00319-4, PMID: 40001253 PMC11852898

